# Values of OAS gene family in the expression signature, immune cell infiltration and prognosis of human bladder cancer

**DOI:** 10.1186/s12885-022-10102-8

**Published:** 2022-09-26

**Authors:** Lijuan Gao, Ruimin Ren, Jing Shen, Jiayi Hou, Junya Ning, Yanlin Feng, Meiyue Wang, Lifei Wu, Yaojun Sun, Huang Wang, Deping Wang, Jimin Cao

**Affiliations:** 1 Key Laboratory of Cellular Physiology, Ministry of Education, Shanxi Medical University, Taiyuan, 030001 China; 2 Department of Physiology, Shanxi Medical University, Taiyuan, 030001 Shanxi Province China; 3 Department of Urology, Shanxi Bethune Hospital (Third Hospital of Shanxi Medical University), Taiyuan, 030032 China; 4 Department of Clinical Laboratory, Shanxi Provincial Academy of Traditional Chinese Medicine, Taiyuan, 030012 China

**Keywords:** OAS gene family, Bladder cancer, Expression signature, Immune cell infiltration, Prognosis

## Abstract

**Background:**

Bladder cancer (BLCA) is one of the most common genitourinary malignancies in the world, but its pathogenic genes have not been fully identified and the treatment outcomes are still unsatisfactory. Although the members of 2', 5'-oligoadenylate synthetase (OAS) gene family are known involved in some tumorous biological processes, the roles of the OAS gene family in BLCA are still undetermined.

**Methods:**

By combining vast bioinformatic datasets analyses of BLCA and the experimental verification on clinical BLCA specimen, we identified the expressions and biological functions of OAS gene family members in BLCA with comparison to normal bladder tissues.

**Results:**

The expression levels of OAS gene family members were higher in BLCA than in normal bladder tissues. The expression levels of most OAS genes had correlations with genomic mutation and methylation, and with the infiltration levels of CD4 + T cells, CD8 + T cells, neutrophils, and dendritic cells in the microenvironment of BLCA. In addition, high expressions of OAS1, OAS2, OAS3, and OASL predicted better overall survival in BLCA patients.

**Conclusions:**

The highly expressed OAS genes in BLCA can reflect immune cells infiltration in the tumor microenvironment and predict the better overall survival of BLCA, and thus may be considered as a signature of BLCA. The study provides new insights into the diagnosis, treatment, and prognosis of BLCA.

**Supplementary Information:**

The online version contains supplementary material available at 10.1186/s12885-022-10102-8.

## Introduction

Bladder cancer (BLCA) is a major malignant tumor in the urinary system, and is one of the most common malignancies worldwide [1]. In 2018, there were nearly 549,000 new BLCA cases and 200,000 deaths worldwide [2]. According to the pathological characteristics, BLCA is classified to non-muscle invasive BLCA (NMIBC) and muscle invasive BLCA (MIBC), with a ratio approximately of 70% and 30%, respectively [3]. Patients with NMIBC have a longer survival period compared with MIBC, although NMIBC is prone to local recurrence and can develop to invasive disease. NMIBC (stage Ta or T1) has a good prognosis with 90% of 5-year overall survival rate, whereas the 5-year overall survival rate of MIBC (stage T2 − T4) decreases to 60% or worse [4, 5], depending on the detrusor muscle invasion [6]. The incidence of BLCA in men is three or four times higher than in women [7], but women are typically diagnosed with more-advanced cancer and have a worse prognosis [8]. Advanced age, male sex, and cigarette smoking are risk factors of BLCA [9]. Cigarette smoking is the most common exposure contributing to the increased incidence of BLCA, and the degree of smoking may relate to the aggressiveness of BLCA [10]. Many BLCA patients are diagnosed at middle or late stage, missing the optimal opportunity for intervention and treatment. Some treatments for BLCA have been developed, including transurethral tumor resection [11], radical cystectomy [9], chemotherapy [12], and bacillus Calmette Guerin treatment [13]. These treatments show great effects in short terms, but the recurrence and metastasis rates are relatively high, and the five-year survival rates are still low. Therefore, exploring the mechanisms of infiltration and metastasis of BLCA and identifying potential therapeutic targets have substantial clinical values.

The 2’, 5’-oligoadenylate synthetase (OAS) family members are IFN-induced antiviral enzymes. OAS family and their downstream effector RNase L play vital roles in host defence against virus infection. In human genome, The OAS family is composed of four members, including OAS1, OAS2, OAS3, and OASL. These four members differ in the numbers of OAS domain, types of synthesized 2–5A, and levels of oligomerization [14–16]. In a previous work focusing on the genetics of psoriasis, we noticed that OAS genes had a strong link with BLCA [17], suggesting that OAS genes might be biological indicators of BLCA. In addition, OAS gene family may contribute to the diagnosis and prognosis of a variety of cancers, including breast cancer [18], pancreatic adenocarcinoma [19], and prostate cancer [20]. However, the potential role of OAS family in BLCA is largely unknown and needs investigation.

In the present study, we comprehensively analyzed the expression characteristics of OAS family in BLCA using multiple bioinformatic databases and approaches, and experimentally verified the bioinformatic results. We demonstrated the value of OAS family in BLCA progression and the preliminary molecular mechanisms. The study may shed lights to the developments of therapeutic strategy and prognosis evaluation for BLCA.

## Materials and methods

### Oncomine dataset analysis

Oncomine gene expression array dataset [21] (https://www.oncomine.org) is an online cancer microarray database, and was used to analyze the transcription levels of OAS family in different cancers. The mRNA expressions of OAS gene family in clinical BLCA specimens were searched and compared with normal bladder specimens. In data processing, we entered all the four OAS genes and selected the cancer type as “bladder cancer”, and let the analysis type be “cancer vs. normal”. The cut-off of *P* value and fold change was defined as 0.0001 and 2, respectively.

### GEPIA dataset analysis

Gene Expression Profiling Interactive Analysis (GEPIA) [22] (http://gepia.cancer-pku.cn/) is a newly developed interactive web server which contains the RNA sequencing expression data of 9,736 malignant tumors from the Cancer Genome Atlas (TCGA) and 8,587 normal samples from the Genotype-Tissue Expression (GTEx) projects. Using GEPIA, we verified the mRNA expression levels of OAS genes in the BLCA tissues and their correlations with the prognosis of BLCA. Box plots, violin plots, dot plots and matrix plots were made using the ‘Expression DIY’ tab of GEPIA. Pairwise gene correlation analyses were performed using the ‘Correlation’ tab based on the given sets of TCGA and/or GTEx expression data. The overall survival associated with OAS genes were analyzed using the ‘Survival’ tab. The corresponding results were obtained after entering the names of genes.

### TIMER analysis

Tumor Immune Estimation Resource (TIMER) [23] (https://cistrome.shinyapps.io/timer/) is a comprehensive resource for systematic analysis of immune infiltrates across diverse cancer types based on 32 cancer types and 10,897 samples from TCGA. In this database, six immune infiltrates, including B cells, CD4 + T cells, CD8 + T cells, neutrophils, macrophages, and dendritic cells, were selected to evaluate the correlation between OAS family and the infiltration of immune cells based on TIMER algorithm. *P* < 0.05 was considered statistically significant.

### Patients and bladder tissue sampling

The study included seven inpatients diagnosed as BLCA in Shanxi Bethune Hospital, Taiyuan, China. Patient information were shown in Table S1. The experiments on patient bladder specimens were mainly used to verify the reliability of bioinformatic analyses. All the patients were in advanced stage and underwent radical tumor excision surgery. The BLCA tissues and paired adjacent normal bladder tissues were harvested during surgeries, and were frozen in liquid nitrogen and stored at an ultra-low-temperature freezer for experiments of qPCR, Western blotting, and immunohistochemistry.

### RNA isolation and quantitative real-time PCR (qPCR)

qPCR was performed to examine the mRNA levels of OAS family in bladder cancer and adjacent tissues. Total RNA was extracted from tissues using TRIzol (Invitrogen, Carlsbad, CA) according to the manufacturer’s instruction. PrimeScript™ RT reagent Kit (TaKaRa, Osaka, Japan) was used to reversely transcript the RNA into cDNA. qPCR was performed according to the instructions of TaKaRa TB Green Premix Ex Taq II (TaKaRa, Osaka, Japan). Primer sets for selected genes were designed by Sangon Biotech Co., Ltd (Shanghai, China). The expression data were normalized to the reference glyceraldehyde-3-phosphate dehydrogenase (GAPDH) and the mRNA levels were calculated using the 2^−ΔΔCt^ method. Primer sequences for qPCR were as follows. OAS1 forward: 5'-AGTTGACTGGCGGCTATAAAC-3', OAS1 reverse: 5'-GTGCTTGACTAGGCGGATGAG-3'. OAS2 forward: 5'-AGGTGGCTCCTATGGACGG-3', OAS2 reverse: 5'-TTTATCGAGGATGTCACGTTGG-3'. OAS3 forward: 5'- GAAGGAGTTCGTAGAGAAGGCG -3', OAS3 reverse: 5'-CCCTTGACAGTTTTCAGCACC-3'. OASL forward: 5'-CCCTTGACAGTTTTCAGCACC-3', OASL reverse: 5'-CTTCAGCTTAGTTGGCCGATG-3'. GAPDH forward: 5'-CTGGGCTACACTGAGCACC-3', GAPDH reverse: 5'-AAGTGGTCGTTGAGGGCAATG-3'.

### Western blotting

We performed the western blotting assay following the methods reported by Wang et al. [24]. Total proteins for Western blotting were extracted from bladder cancer tissues and adjacent normal bladder tissues, respectively. The protein concentrations in all samples were quantified using the bicinchoninic acid (BCA) assay (Solarbio Co., Ltd, Beijing, China). A total amount of 40 μg extracted protein from each sample were separated by 10% SDS-PAGE. Then, proteins from the SDS-PAGE gel were transferred to polyvinylidene fluoride (PVDF) membrane (Millipore, Billerica, MA, USA). Membranes were blocked with 5% nonfat milk for 1 h at room temperature. The membranes were incubated with respective primary antibodies for overnight at 4 °C. The membranes were washed with TBST and then incubated with the secondary antibody conjugated with horseradish peroxidase for 1 h at room temperature. Target immunoreactive bands were visualized with enhanced chemiluminescent substrate (Boster Biological Technology, Wuhan, China). The gray values of protein bands were determined using Image Lab 2.0 (Genmall Biotechnology Co.,Ltd, Wuhan, China), and β-actin (ZSGB-Bio, China) was used for normalization. The primary antibodies (anti-OAS1, OAS2, and OAS3) were purchased from Peprotech (New Jersey, USA), anti-OASL was purchased from Abcam (Cambridge, MA, USA), the secondary antibodies were purchased from Zhongshan Golden bridge Biotechnology (Beijing, China).

### Immunohistochemistry

To perform immunohistochemical staining of OAS1, OAS2, OAS3, and OASL, bladder cancer tissues and adjacent normal bladder tissues were fixed in 10% formalin, embedded in paraffin, sectioned (3 μm) and attached to slides. Tissue sections were incubated with commercial rabbit polyclonal antibodies against OAS1 (dilution 1:300), OAS2 (dilution 1:300), OAS3 (dilution 1:100), and OASL (dilution 1:250) overnight at 4 °C. Then, the sections were conjugated with a horseradish peroxidase (HRP) antibody (dilution 1:500) at room temperature for 2 h, washed with PBS, reacted with 3,3-diaminobenzidine (DAB), then washed with water, counterstained, and cover-slipped. Positive staining signals of all fields were observed under a light microscope and images were taken.

### UALCAN

UALCAN [25, 26] (http://ualcan.path.uab.edu/) is a user-friendly comprehensive web resource for analyzing the RNA sequencing expression data from TCGA. Here, UALCAN was used to analyze the relationship between OAS gene expression and tumor stage. In this analysis, we selected bladder urothelial carcinoma in TCGA to get OAS gene expression information based on "Individual cancer stages".

### Kaplan–Meier plotter and Oncolnc analysis

The Kaplan–Meier (KM) plotter [27] (https://kmplot.com/analysis/) can be used to analyze the survival biomarkers across 21 cancer types, based on sources including Gene Expression Omnibus database (GEO), European Genome-phenome Archive (EGA), and TCGA. Here, KM plotter was used to estimate the value of OAS gene family in predicting the overall survival (OS).

OncoLnc [28] (http://www.oncolnc.org/) is a newly available tool for interactively exploring survival correlations and for downloading clinical data coupled to gene expression data. It contains the survival data of 8,647 patients from 21 cancer studies in TCGA, and can create high quality OS plots. Here, OncoLnc was used to perform survival analysis in BLCA.

### cBioPortal analysis

The cBio Cancer Genomics Portal [29] (cBioPortal, https://www.cbioportal.org/) is a comprehensive open-access web resource which can help to visualize and explore multidimensional cancer genomics data. Using cBioPortal database, we analyzed the genetic alterations of OAS gene family in BLCA, including mutation and methylation. We chose 12 studies of bladder urothelial carcinoma, and entered OAS1, OAS2, OAS3 and OASL to search for the information. Genetic alterations, including mutation and methylation of OAS gene family in BLCA, were analyzed using the "OncoPrint", "Cancer Types Summary", "plots", and "Mutations" tabs.

### GeneMANIA analysis

GeneMANIA [30] (http://www.genemania.org) is a user-friendly website that provides information for protein and genetic interactions, pathways, co-expression, co-localization, and protein domain similarity. Here, the top 20 closely related neighbor genes of OAS family were identified using GeneMANIA.

### Gene Ontology (GO) enrichment analysis

DAVID 6.8 [31] (https://david.ncifcrf.gov/home.jsp) is a comprehensive, functional annotation website that clarify the biological function of submitted genes and can be used for GO analysis. Using DAVID 6.8 online software, we identified the top 20 closely related neighbor genes of OAS family. Biological processes (BP), cellular components (CC), and molecular functions (MF) were determined using the GO enrichment analysis. Enrichment results were visualized using the R project (v3.5.3) “ggplot2” package. *P* < 0.05 was considered statistically significant.

### Kyoto Encyclopedia of Genes and Genomes (KEGG) pathway enrichment analysis

KEGG [32] (https://www.kegg.jp/) is an online database for systematic analysis of gene function and genomic information, which integrate information from genomics, biochemistry, and functional omics. KEGG pathway is one of 16 sub-databases that contains different types of information, including molecular interactions and relationship networks related to metabolism, regulation, pathways, biochemistry, disease, and drugs. KOBAS 3.0 [33] (http://kobas.cbi.pku.edu.cn/anno_iden.php) online software was used to analyze the KEGG pathway and the enrichment results were visualized with the R project (v3.5.3) “ggplot2” package. *P* < 0.05 was considered statistically significant.

### Statistical analysis

All statistical analyses were performed using GraphPad Prism 5.0 software. Data were represented as the mean ± standard deviation (SD). Two-tail *t*-test was used to compare the means of two sample groups. Statistical significance was set at *P* < 0.05.

## Results

### Transcriptional levels of OAS family in different types of human cancers

**Fig. 1:**
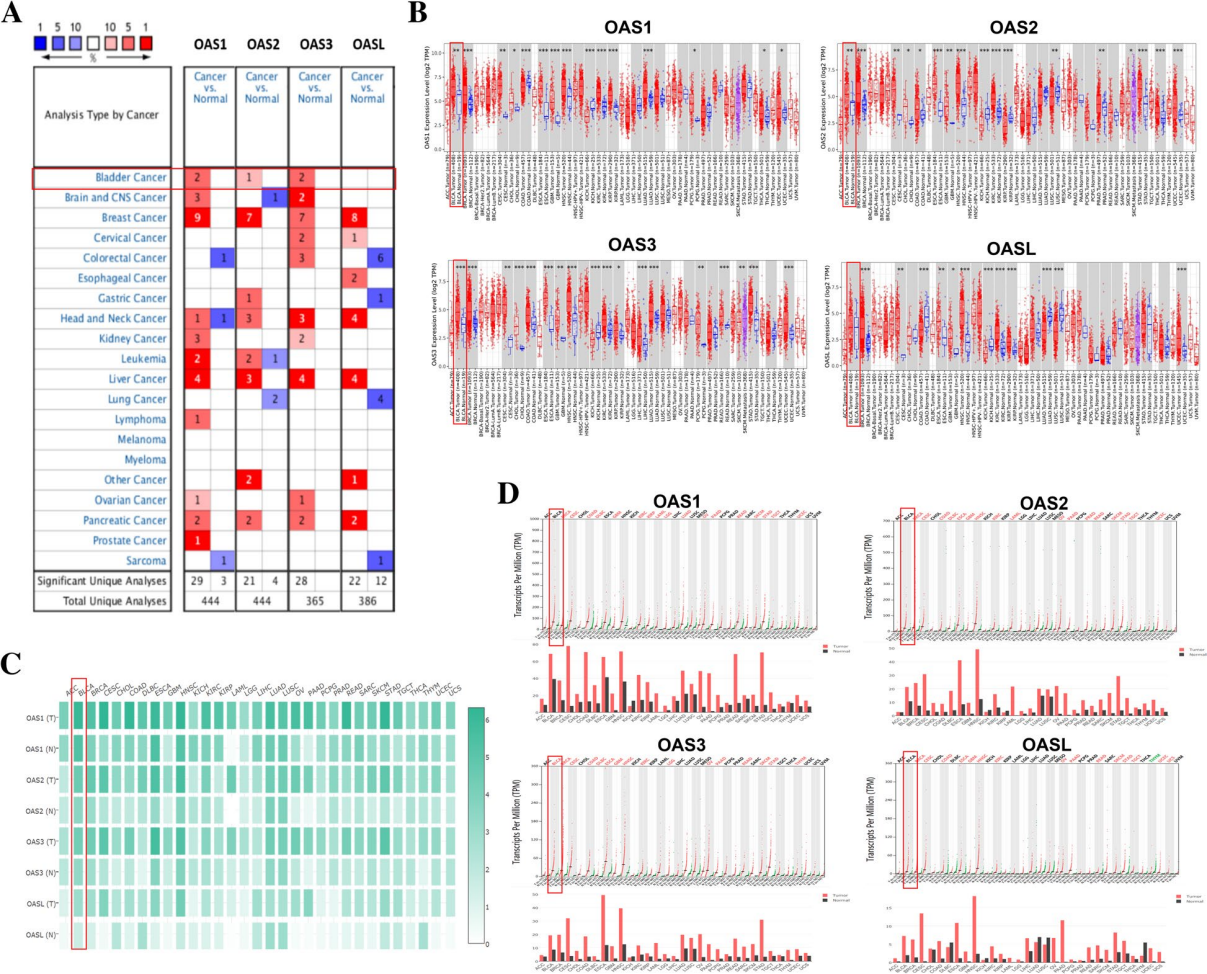
The mRNA expression patterns of OAS1, OAS2, OAS3, and OASL in different types of human cancer. **A** Results of Oncomine database analysis. Red colored squares at the left column indicated higher expression (tumor vs. normal), while blue colored squares at the right column indicated lower expression (tumor vs. normal). The numbers in the red squares represented evidence amounts of higher expression. The numbers in blue squares indicated evidence amount of lower expression. Color shades indicated expression levels. White squares referred to no evidence or no change in expression (Fold change > 2, *P* < 0.0001). **B** Expression levels of OAS gene family in different types of human tumors derived from TCGA database and determined by TIMER. BLCA was marked by a red square in each subpanel (* *P* < 0.05, ** *P* < 0.01, *** *P* < 0.001). **C** Expressions of the four OAS genes in various cancers from GEPIA. Color depth represented expression level. BLCA was marked by a red square. T: tumor. N: normal. **D** Results of GEPIA database analysis. Red fonts represented significantly high expression; green fonts meant low expression; black fonts denoted undifferentially expressed OAS genes in these cancers. Abbreviations of tumor names: ACC, adrenocortical carcinoma. BLCA, bladder cancer. BRCA, breast invasive carcinoma. CESC, cervical squamous cell carcinoma and endocervical adenocarcinoma. CHOL, cholangio carcinoma. COAD, colon adenocarcinoma. DLBC, lymphoid neoplasm diffuse large B-cell lymphoma. ESCA, esophageal carcinoma. GBM, glioblastoma multiforme. HNSC, head and neck squamous cell carcinoma. KICH, kidney chromophobe. KIRC, kidney renal clear cell carcinoma. KIRP, kidney renal papillary cell carcinoma. LAML, acute myeloid leukemia. LGG, brain lower grade glioma. LIHC, liver hepatocellular carcinoma. LUAD, lung adenocarcinoma. LUSC, lung squamous cell carcinoma. OV, ovarian serous cystadenocarcinoma. PAAD, pancreatic adenocarcinoma. PCPG, pheochromocytoma and paraganglioma. PRAD, prostate adenocarcinoma. READ, rectum adenocarcinoma. SARC, sarcoma. SKCM, skin cutaneous melanoma. STAD, stomach adenocarcinoma. TGCT, testicular germ cell tumor. THCA, thyroid carcinoma. THYM, thymoma. UCEC, uterine corpus endometrial carcinoma. UCS, uterine carcinosarcoma. UVM, uveal melanoma

To determine the difference of OAS gene family expression levels between tumor tissues and normal tissues, we identified the mRNA levels of OAS family in various cancers based on Oncomine database, TIMER database, and GEPIA database. Analysis of Oncomine showed that the OAS family was highly expressed in various types of cancers including breast cancer, liver cancer, pancreatic cancer, and bladder cancer, compared with respective normal tissues. From the color shade and the number of evidences, we observed that the expression of OAS gene family in bladder cancer was increased, although this increase was less prominent than in breast cancer (Fig. 1A). This result suggested a potential role of OAS genes in bladder cancer. We further analyzed the TIMER and GEPIA databases. Results showed that OAS genes were all highly expressed in bladder cancer compared with the respective normal tissues (Fig. 1B−D). These results strengthened the value of OAS genes in bladder cancer and inspired us to perform the following experiments.

### Detailed mRNA expressions of OAS family in BLCA and normal bladder tissues and verification by qPCR, Western blotting, and immunohistochemistry

Analyses of Oncomine and GEPIA databases and qPCR experiments were performed to demonstrate the detailed mRNA expression levels of OAS family in BLCA and normal bladder tissues. Oncomine analysis showed that expressions of all the four OAS members, including OAS1, OAS2, OAS3, and OASL, were significantly upregulated in BLCA tissues compared with the normal tissues. Oncomine analysis showed that the mRNA levels of OAS family in BLCA tissues were higher than in normal bladder tissues mainly in three datasets, including Sanchez-Carbayo Bladder 2 [34], Dyrskjot Bladder 3 [35], and Lee Bladder [36] datasets, with |logFC|> 1 (FC, fold change) and *P* < 0.05 (Table 1).

**Table 1:** The mRNA levels of OAS gene family in different types of BLCA tissues and normal bladder tissues (from Oncomine)

**Gene**	**Types of Bladder Cancer vs. Normal**	**Fold change**	***P*-value**	**t-test**	**Data source**
OAS1	Superficial Bladder Cancer (28) vs. Normal (48)	8.816	6.52E-18	11.253	Sanchez-Carbayo Bladder 2
	Infiltrating Bladder Urothelial Carcinoma (81) vs. Normal (48)	2.755	1.59E-9	6.427	Sanchez-Carbayo Bladder 2
	Superficial Bladder Cancer (28) vs. Normal (14)	2.344	3.08E-4	3.889	Dyrskjot Bladder 3
OAS2	Infiltrating Bladder Urothelial Carcinoma (81) vs. Normal (48)	2.470	1.32E-7	5.621	Sanchez-Carbayo Bladder 2
	Superficial Bladder Cancer (126) vs. Normal (68)	1.435	8.25E-4	3.200	Lee Bladder
OAS3	Infiltrating Bladder Urothelial Carcinoma (81) vs. Normal (48)	2.284	6.01E-10	6.667	Sanchez-Carbayo Bladder 2
	Superficial Bladder Cancer (28) vs. Normal (48)	3.675	2.96E-15	9.908	Sanchez-Carbayo Bladder 2
	Infiltrating Bladder Urothelial Carcinoma (13) vs.Normal (14)	1.567	0.003	3.185	Dyrskjot Bladder 3
	Superficial Bladder Cancer (28) vs. Normal (14)	1.380	6.78E-4	3.461	Dyrskjot Bladder 3
OASL	Infiltrating Bladder Urothelial Carcinoma (81) vs. Normal (48)	1.455	4.31E-7	5.204	Sanchez-Carbayo Bladder 2

OAS1 was overexpressed with a fold change of 8.816 in superficial bladder cancer, and with a fold change of 2.755 in infiltrating bladder urothelial carcinoma, in the Sanchez-Carbayo bladder 2 dataset. OAS1 was also highly expressed in superficial bladder cancer (fold change = 2.344) in the Dyrskjot bladder 3 dataset (Table 1).

OAS2 was 2.470 times higher in infiltrating bladder urothelial carcinoma in the Sanchez-Carbayo bladder 2 dataset, and was 1.435 times higher than with the respective normal tissues in superficial bladder cancer from the Lee Bladder dataset (Table 1).

OAS3 was also upregulated in infiltrating bladder urothelial carcinoma and superficial bladder cancer with a fold change respectively of 2.284 and 3.675 in the Sanchez-Carbayo bladder 2 dataset. In the Dyrskjot bladder 3 dataset, OAS3 was 1.567 times higher in infiltrating bladder urothelial carcinoma and was 1.380 times higher in superficial bladder cancer than in the respective normal tissues (Table 1).

OASL was only reported in the Sanchez-Carbayo bladder 2 dataset and was 1.455 times higher in infiltrating bladder urothelial carcinoma than in the normal samples (Table 1).

**Fig. 2:**
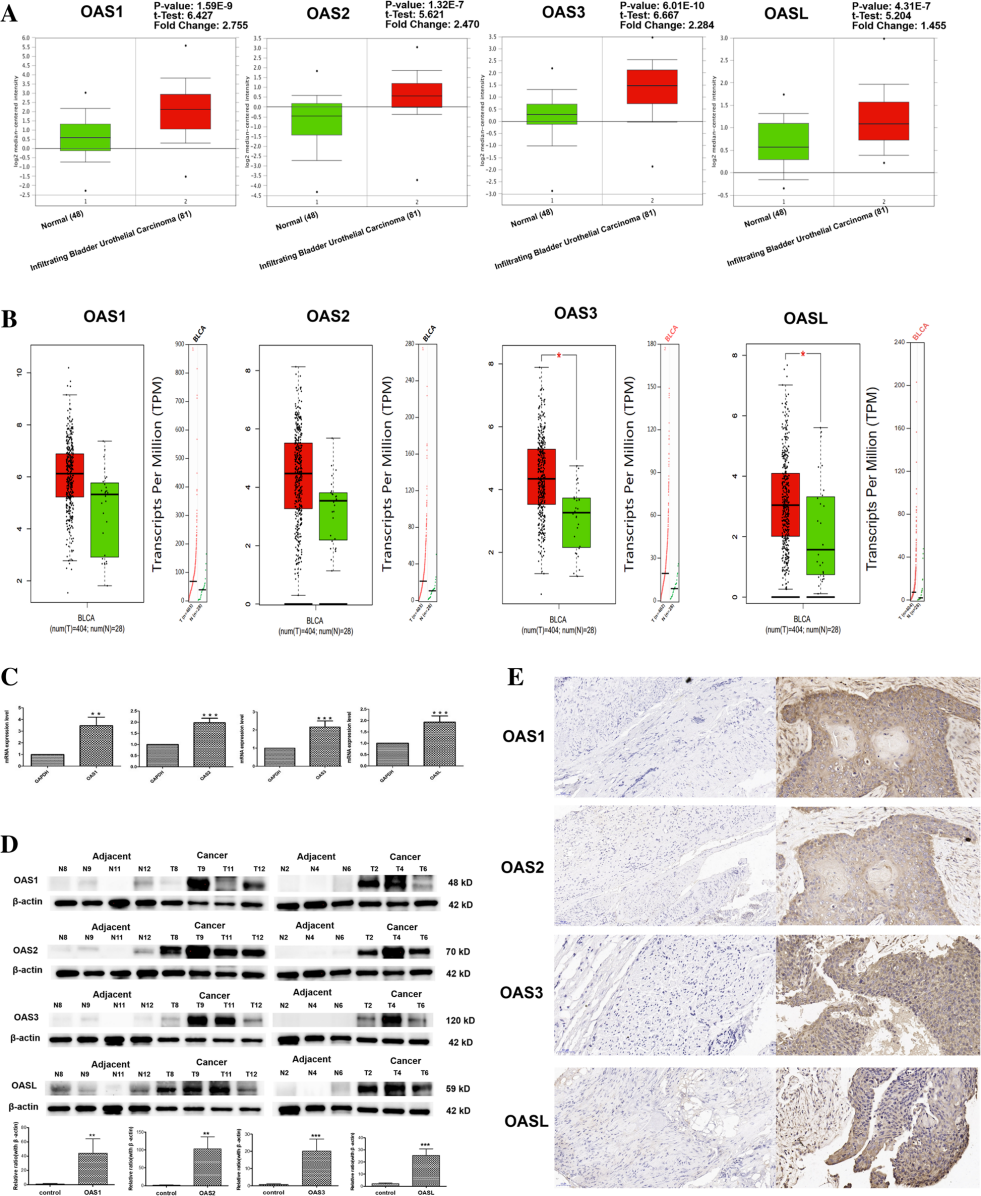
Detailed expression levels of OAS gene family in bladder cancer. **A** Box plot of OAS1, OAS2, OAS3, and OASL expression levels in Sanchez-Carbayo Bladder 2 dataset from Oncomine. Red meant normal tissues, and green meant infiltrating bladder urothelial carcinoma tissues. **B** Expression characterization of OAS1, OAS2, OAS3, and OASL in GEPIA database. Red and green represented tumor and normal tissues, respectively. **C** Results of qPCR showing the mRNA levels of OAS family in BLCA tissues and normal bladder tissues. * *P* < 0.05, ** *P* < 0.01, *** *P* < 0.001, *n* = 7 samples for each group. **D** Results of Western blotting. Upper, representative electrophoresis bands of Western blots for OAS family protein expressions in BLCA tissues and normal bladder tissues. Lower, statistical results of Western blots. * *P* < 0.05, ** *P* < 0.01, *** *P* < 0.001, *n* = 7 samples for each group. **E** Immunohistochemical stains of OAS family proteins in BLCA and normal tissues. Brown colors represented positive staining signals. It is obvious that all the four OAS genes were highly expressed in BLCA tissues compared with the normal bladder tissues

To better characterize the transcription levels of OAS gene family in BLCA, we selected some representative results of Sanchez-Carbayo Bladder 2 dataset analysis and showed in Fig. 2A, which showed that all the mRNA levels of the four OAS genes were upregulated in BLCA compared with the normal tissues. The fold change of OAS1, OAS2, OAS3, and OASL was 2.755, 2.470, 2.284 and 1.455, respectively. In this analysis, the fold change of OAS1 was highest.

Figure 2B showed the mRNA levels of the four OAS genes obtained from GEPIA database analysis, the mRNA expressions of OAS1, OAS2, OAS3, and OASL were all higher in BLCA tissues than in the normal tissues, especially the expression of OAS3 and OASL is significant (cut off: |logFC|> 1 and q-value < 0.01).

To further verify the bioinformatic results shown in Fig. 1, Fig. 2A, B and Table 1, we performed qPCR, Western blotting, and immunohistochemistry on human BLCA tissues and paired adjacent normal bladder tissues. qPCR results were consistent with bioinformatic results (Fig. 2C). Western blotting results showed that the protein expression levels of OAS1, OAS2, OAS3, and OASL in BLCA were all significantly elevated in BLCA compared with the normal tissues (Fig. 2D). Immunohistochemical stains were consistent with the results of bioinformatics, qPCR, and Western blotting (Fig. 2E). Overall, both database analysis and experimental validation have proved that the OAS family is highly expressed in BLCA.

### Values of OAS genes expression in tumor staging and prognosis of BLCA

**Fig. 3:**
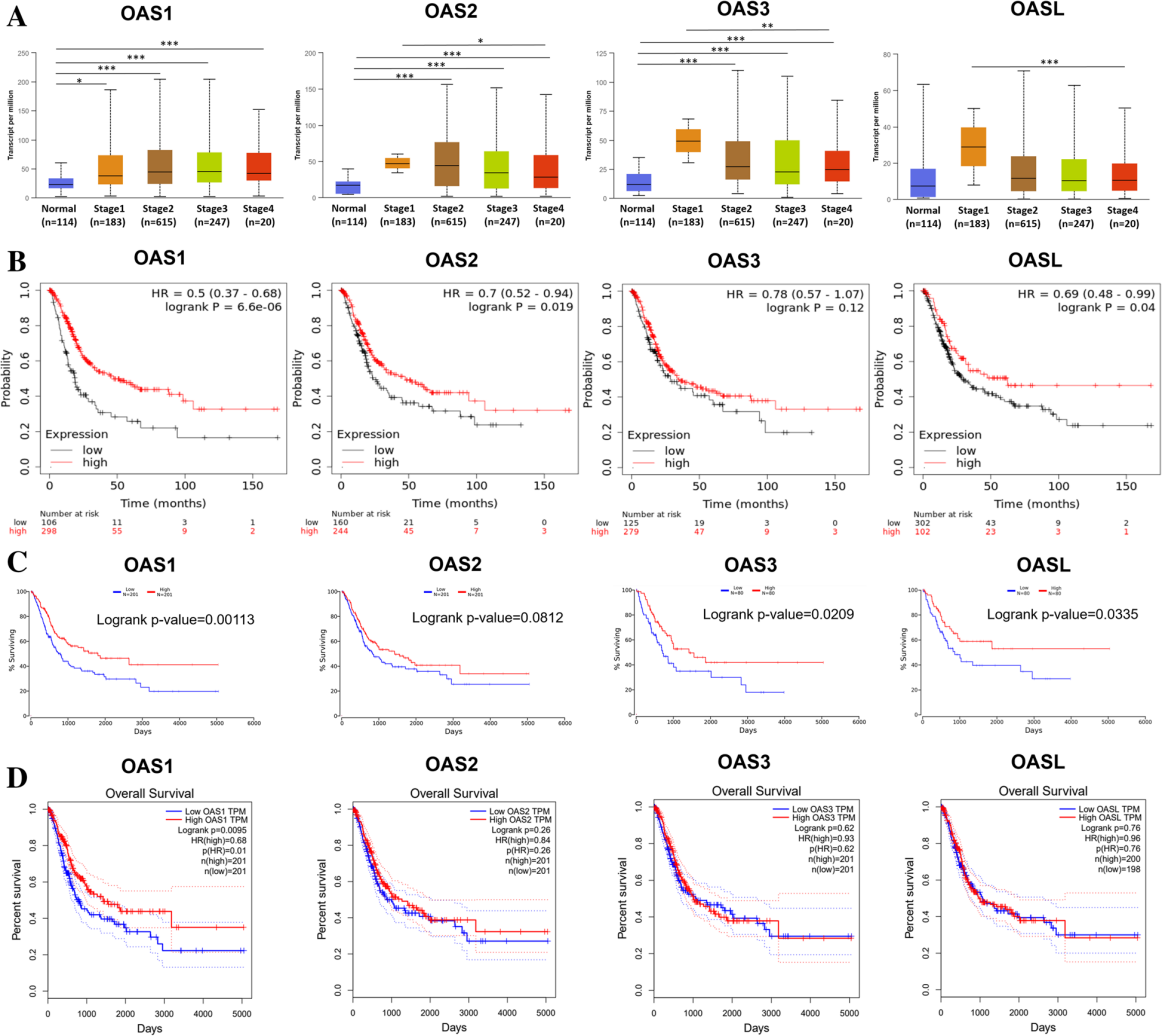
**A** The relationship between OAS gene expression and the clinical stage of BLCA. **B-D** Prognostic values of OAS genes in predicting the survival of BLCA. Data were derived from Kaplan–Meier Plotter, OncoLnc, and GEPIA database

For further validation, we analyzed the expressions of OAS1, OAS2, OAS3, and OASL in various clinical stages for BLCA using UALCAN. Results indicated that OAS1 expression kept a high level in various clinical stages (stages 1 − 4) of BLCA. OAS2 and OAS3 were also high in stages 1 − 4, but they were the highest at stage 2, then declined at stages 3 − 4 compared to stage 1. The expression of OASL in different stages showed a similar trend as OAS2 or OAS3, but did not reach a statistical significance (Fig. 3A).

The potential prognostic values of OAS1, OAS2, OAS3, and OASL in BLCA were investigated using Kaplan–Meier Plotter, OncoLnc, and GEPIA. Results indicated that increased levels of OAS1, OAS2, OAS3, and OASL were associated with better overall survival (OS) in BLCA. Especially, higher mRNA levels of OAS1, OAS2, and OASL were significantly related to greater OS in Kaplan–Meier Plotter (Fig. 3B), and higher mRNA levels of OAS1, OAS3, and OASL were significantly related to greater OS in OncoLnc (Fig. 3C). Nevertheless, prognostic analyses of GEPIA database showed that only increased OAS1 mRNA was associated with favorable OS in BLCA (Fig. 3D). Thus, OAS1 might be a prognostic indicator for BLCA.

### Relation between OAS family expression and immune cells infiltration

**Fig. 4:**
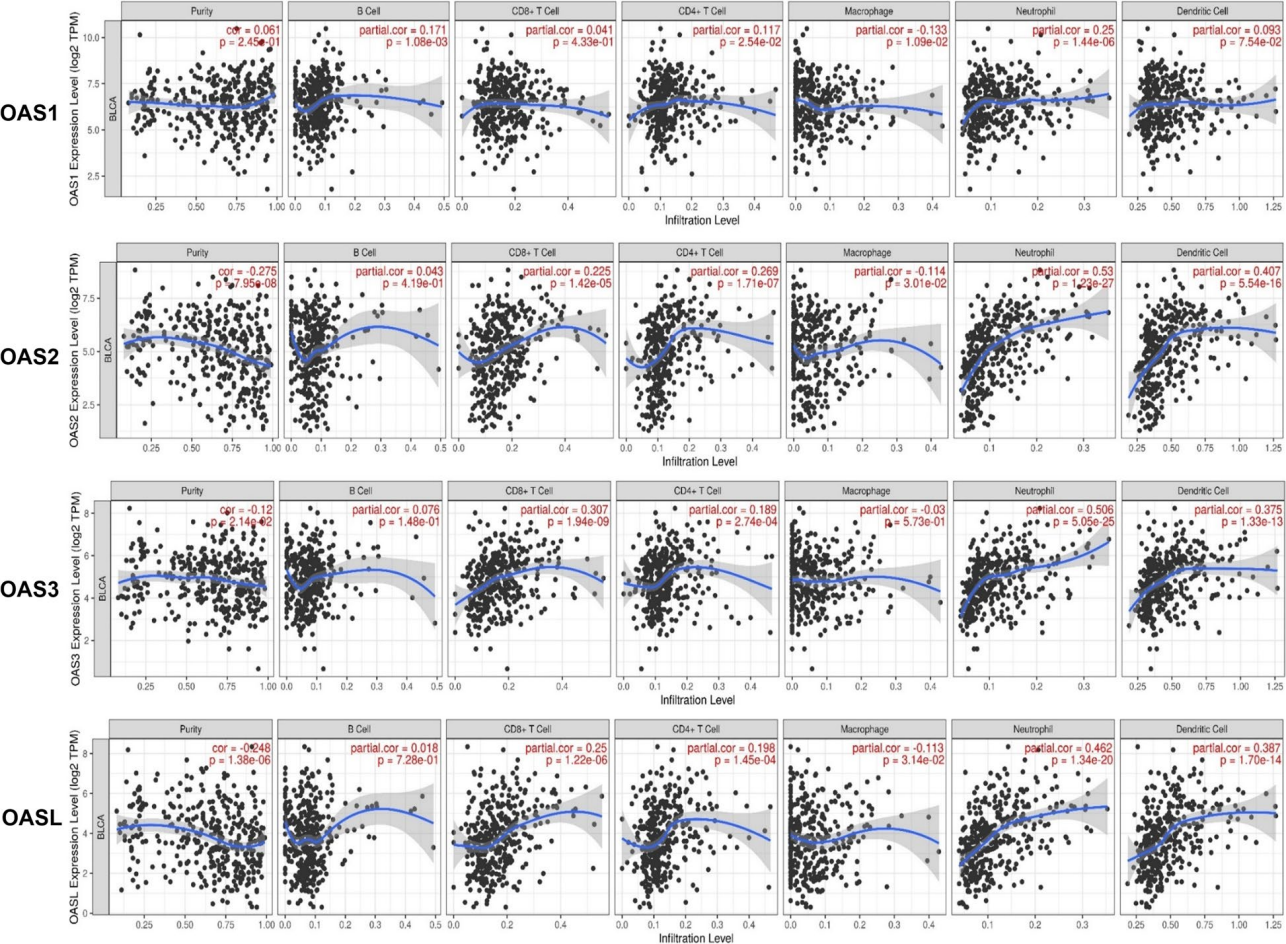
Correlation of OAS gene family with immune cell infiltration level in BLCA derived from Tumor Immune Estimation Resource (TIMER). The horizontal axis represented the infiltration levels of tumor immune cells. The vertical axis indicated the expression levels of OAS genes. Cor. > 0 indicated the positive correlation of OAS family expression level with tumor immune cell infiltration. Cor. < 0 indicated the negative correlation of OAS family expression level with tumor immune cell infiltration. OAS1 expression had no significant correlation with tumor purity but had significant positive correlations with infiltrating levels of B cells, CD4 + T cells, neutrophils, and dendritic cells in BLCA. OAS2 expression had positive correlations with the infiltrating levels of CD8 + T cells, CD4 + T cells, neutrophils, and dendritic cells, while had negative correlations with tumor purity and the infiltrating level of macrophages in BLCA. OAS3 expression was negatively corelated with tumor purity and had positive correlations with the infiltrating levels of CD8 + T cells, CD4 + T cells, neutrophils, and dendritic cells in BLCA. OASL expression had positive correlations with the infiltrating levels of CD8 + T cells, CD4 + T cells, neutrophils, and dendritic cells, but had negative correlations with tumor purity and macrophage infiltration in BLCA

Tumor microenvironment (TME) [37] is a complex milieu of immune cells, connective tissue cells, and vascular components that are essential to cancer progression and metastasis. Previous studies have shown that the prognosis and therapeutic response of cancer were closely related to TME, especially the tumor-infiltrating immune cells. We investigated whether OAS family expression was associated with the level of immune cell infiltration in BLCA using TIMER database (Fig. 4). Results showed that the expressions of OAS2, OAS3, and OASL had significant negative correlations with tumor purity in BLCA. OAS1 expression showed positive correlation with the infiltration level of B cells (partial.cor = 0.171), and other three OAS genes did not have significant correlation with B cells. OAS2, OAS3, and OASL had positive correlations with the infiltration levels of CD8 + T cells, with the partial correlations (partial.cor) respectively of 0.225, 0.307, and 0.25. All the four OAS genes had significant correlations with CD4 + T cells infiltration (partial.cor = 0.117, 0.269, 0.189, and 0.198, respectively for OAS1, OAS2, OAS3, and OASL). OAS1, OAS2, and OASL had negative correlations with macrophage infiltration (partial.cor =  − 0.133, − 0.114, − 0.113, respectively). All the four OAS family had significant correlations with the infiltrations of neutrophils and dendritic cells. The partial correlations of OAS1, OAS2, OAS3, and OASL with neutrophils were respectively 0.25, 0.53, 0.506, and 0.462. The partial correlations of OAS1, OAS2, OAS3, and OASL with dendritic cells were respectively 0.093, 0.407, 0.375, and 0.387.

### Genetic and epigenetic changes of OAS gene family in BLCA

**Fig. 5:**
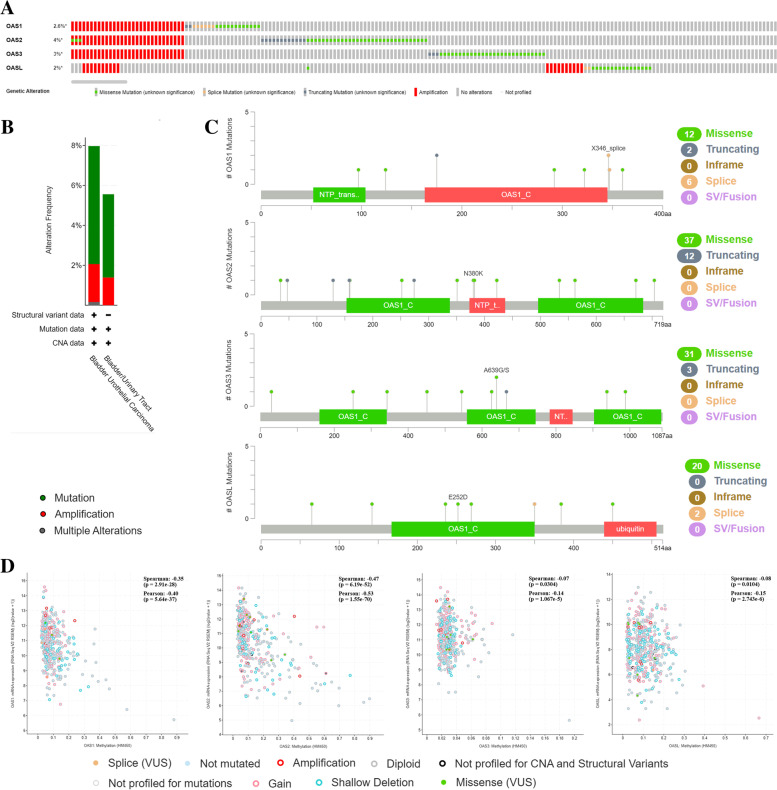
Visual summary of OAS gene family alterations in BLCA. **A** OAS gene family expression and mutation analysis in BLCA. **B** Frequency of gene alterations in OAS1, OAS2, OAS3, and OASL in different types of BLCA. **C** Mutational locations of OAS1, OAS2, OAS3, and OASL genes. **D** Effects of DNA methylation on the mRNA expressions of OAS genes

To determine the frequency and type of OAS gene family alterations in BLCA, we analyzed the mutation and methylation of OAS genes in the BLCA dataset using cBioPortal based on 2,365 patients/2,410 samples of 12 studies. Results demonstrated that patient numbers (percentages) showing genetic alterations of OAS1, OAS2, OAS3, and OASL were respectively 50 (2.5%), 74 (4%), 61 (3%), and 39 (2%) (Fig. 5A). In addition, the mutation frequencies of OAS gene family were 5.56% and 7.97% respectively in the bladder/urinary tract subtype and bladder urothelial carcinoma subtype (Fig. 5B). Genetic mutations of OAS gene family mainly showed missense, truncating, and splicing (Fig. 5C). Furthermore, the degrees of DNA methylation of OAS gene family were negatively correlated with the expression level of OAS family (Fig. 5D), suggesting that DNA methylation may suppress the transcription of the OAS genes.

### Relationships among OAS family and co-expressed genes of OAS family in BLCA

**Fig. 6:**
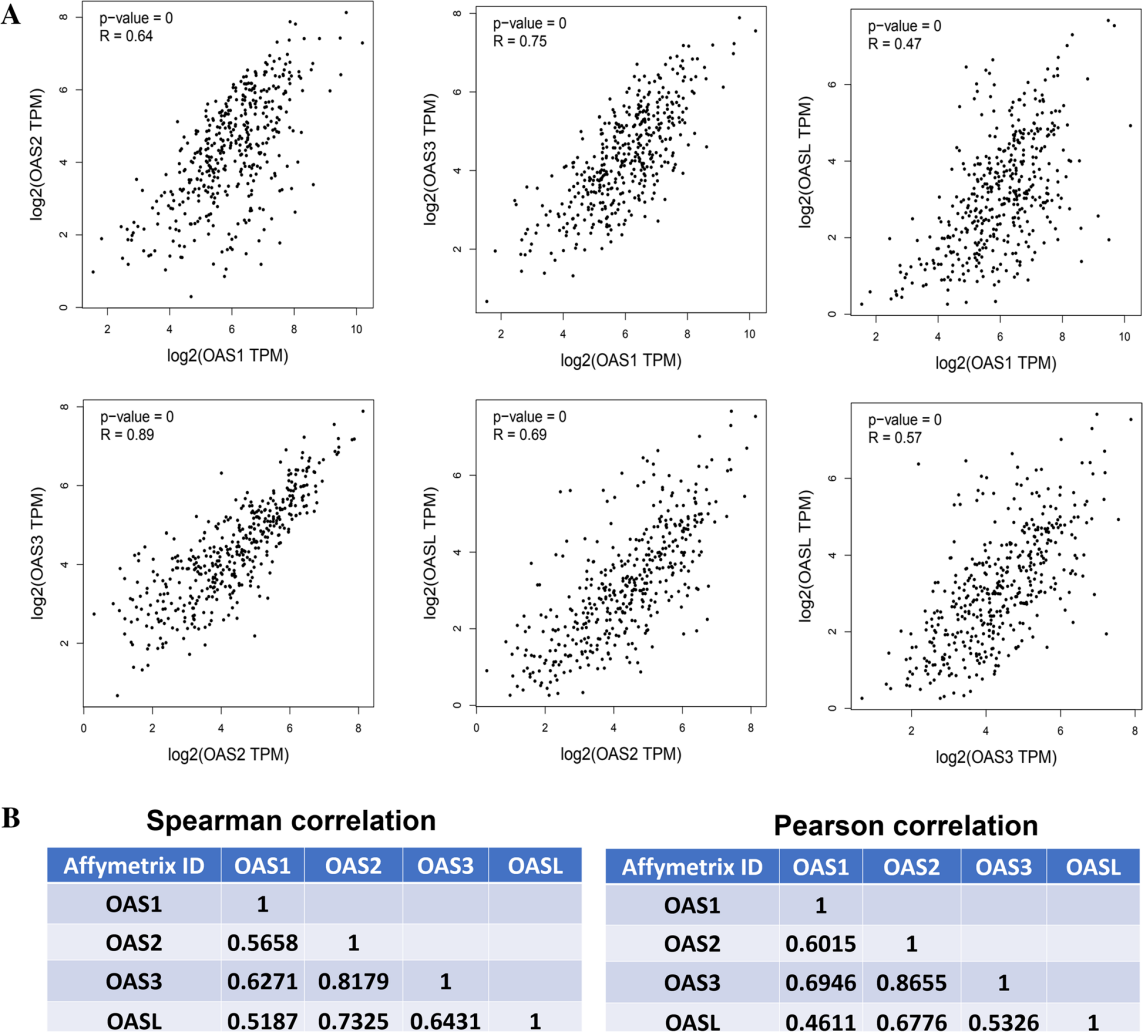
Relationship among the OAS genes. **A** Pearson correction analysis of GEPIA database. **B** Spearman and Pearson correction analysis among the OAS genes. Data were derived from Kaplan–Meier Plotter

The OAS family members may cooperate in the development of BLCA. Therefore, we investigated the potential correlations among OAS1, OAS2, OAS3, and OASL. OAS1 expression level was positively correlated with OAS2, OAS3, OASL (*R* = 0.64, 0.75, and 0.47, respectively) in the GEPIA database. The expression of OAS2 was positively correlated with OAS3 and OASL (*R* = 0.89 and 0.69, respectively), and OAS3 expression was positively correlated with OASL (*R* = 0.57) in GEPIA database analysis (Fig. 6A). Similar results were obtained from a Kaplan–Meier Plotter analysis (Fig. 6B). These correlation analyses indicated that the four OAS genes were positively correlated with each other which may reflect the collaboration of the four genes in BLCA.

**Fig. 7:**
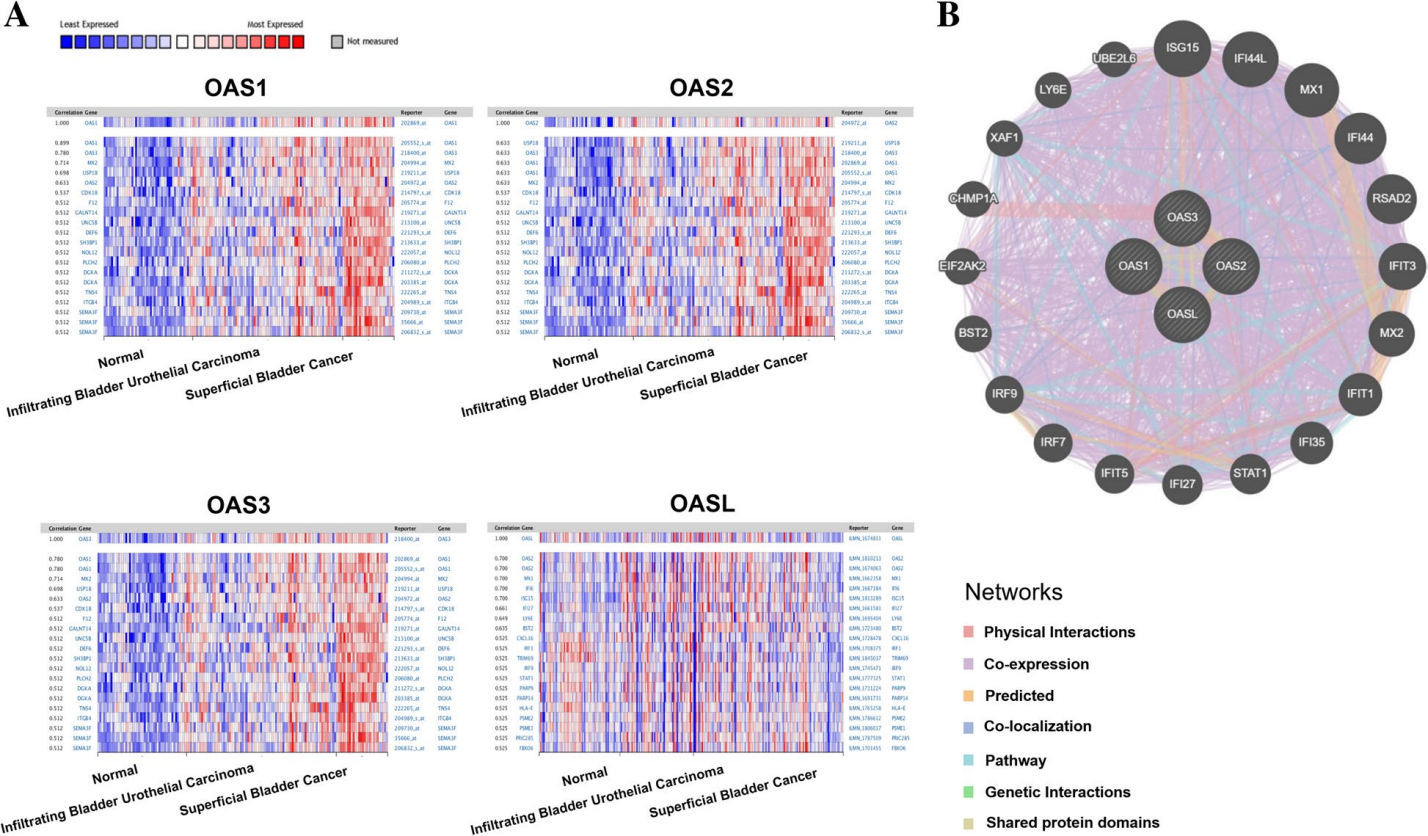
Co-expressed genes of OAS1, OSA2, OAS3, and OASL. **A** The genes relevant to the expressions of OAS1, OSA2, OAS3, and OASL, respectively. **B** The common network for OAS genes and their neighboring top 20 co-expressed genes

The co-expressed genes of OAS family in BLCA were examined using the Oncomine and GeneMANIA databases. The genes co-expressed with OAS1, OAS2, OAS3, and OASL in Sanchez-Carbayo Bladder 2 dataset were identified using Oncomine in 48 normal samples, 81 filtrating bladder urothelial carcinoma tissues, and 28 superficial bladder cancer tissues. Results showed that OAS1 was positively correlated with OAS3, MX2, USP18, F12, GAALNT14, UNC58, DEF6, SH3BP1, NOL12, PLCH2, DGKA, THS4, ITGB4, and SEMA3F. OAS2 was positively correlated with USP18, OAS3, OAS1, MX2, CDK18, F12, GALNT14, UNC5B, DEF6, SH3BP1, NOL12, PLCH12, DGKA, TNS4, ITGB4, and SEMA3F. OAS3 was positively correlated with OAS1, MX2, USP18, OAS2, CDK18, F12, GALNT14, UNC5B, DEF6, SH3BP1, NOL12, PLCH2, DGKA, TNS4, ITGB4, and SEMA3F. OASL was positively associated with OAS2, MX1, IF16, ISG15, IF127, LY6E, BST2, CXCL16, IRF1, TRIM69, IRF9, STAT1, RARP9, PARP14, HLA-E, PSME2, PAME1, PR1C285, and FBXO6 (Fig. 7A). Analysis of GeneMANIA showed that the top 20 co-expressed genes of OAS family included ISG15, IFI4L, MX1, IFI44, RSAD2, IFIT3, MX2, IFIT1, IFI35, STAT1, IFI27, IFIT5, IRF7, IRF9, BST2, EIF2AK2, CHMP1A, XAF1, LY6E, and UBE2L6 (Fig. 7B). These top 20 co-expression genes were chosen to perform further biological function analysis (shown below).

### Biological functions and pathways of OAS family in BLCA

**Fig. 8:**
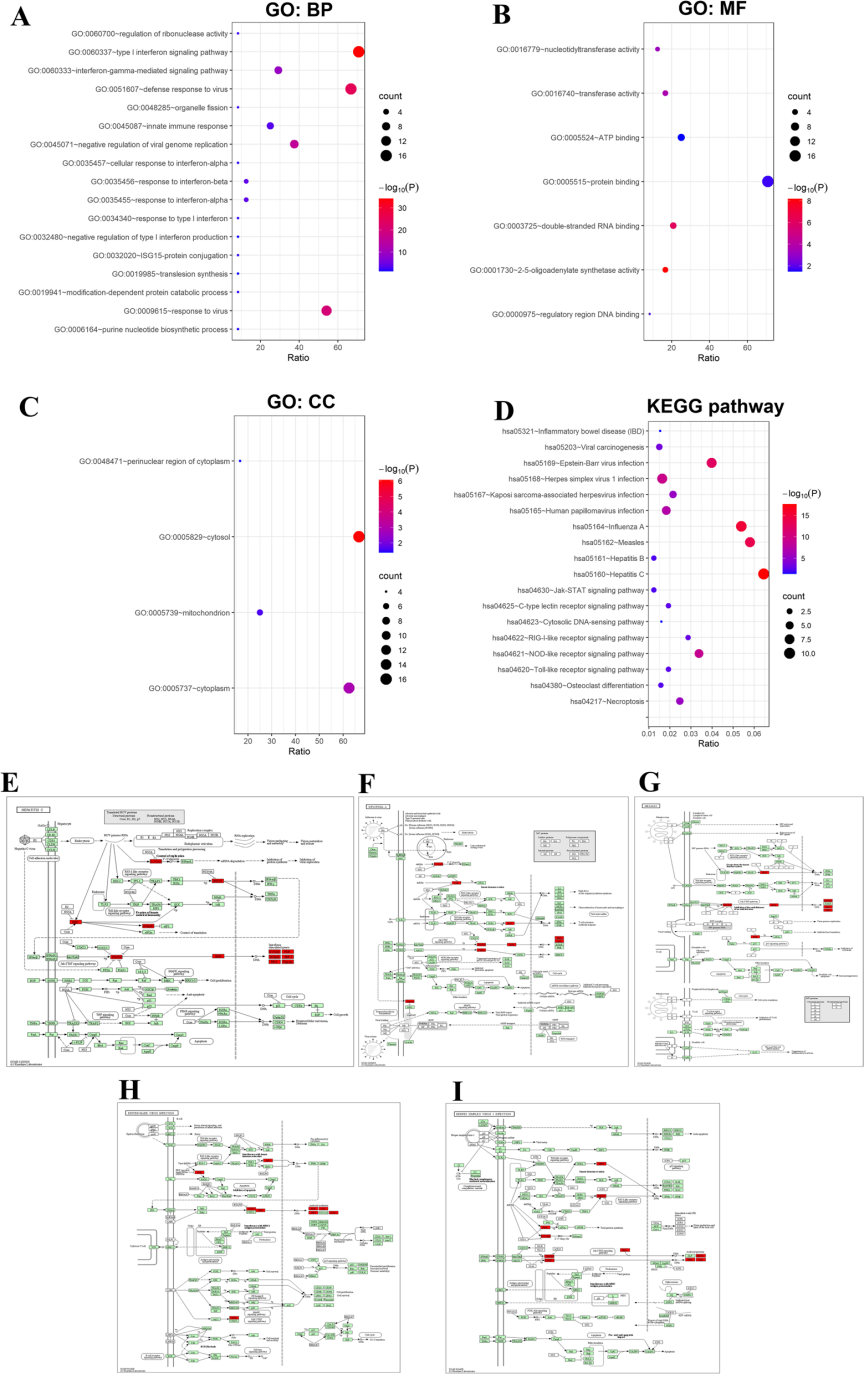
GO and KEGG pathway enrichment analyses of OAS gene family (*P* < 0.05). **A-C** GO enrichment analysis predicted the functional roles of OAS genes based on three aspects, including biological processes, cellular components, and molecular functions. **D** KEGG pathway analysis. **E-I** Top 5 KEGG pathways of OAS gene family and top 20 co-expressed genes. The top 5 KEGG pathways included hepatitis C **E**, influenza A **F**, measles **G**, Epstein-Barr virus infection **H**, and herpes simplex virus 1 infection **I**

GO enrichment analysis predicted the functional roles of target host genes based on three aspects, including biological processes (BP), cellular components (CC), and molecular functions (MF). We found that GO:0,060,337 (type I interferon signaling pathway), GO:0,051,607 (defense response to virus), GO:0,009,615 (response to virus), GO:0,045,071 (negative regulation of viral genome replication), GO:0,060,333 (interferon-gamma-mediated signaling pathway), GO:0,001,730 (2'-5'-oligoadenylate synthetase activity), GO:0,003,725 (double-stranded RNA binding), and GO:0,005,829 (cytosol) played critical roles in the development and progression of BLCA. Most of these functions were related to the process of virus infection and immune response, indicating the correlation between immune cell infiltration and tumorigenesis (Fig. 8A−C; Tables S2, S3 and S4).

By analyzing the KEGG pathway, we identified 21 pathways related to the functions of OAS family and neighbor genes in the pathogenesis of BLCA, including hsa05160 (Hepatitis C), hsa05164 (Influenza A), hsa05162 (Measles), hsa05169 (Epstein-Barr virus infection), hsa05168 (Herpes simplex virus 1 infection), hsa04621 (NOD-like receptor signaling pathway), hsa05165 (Human papillomavirus infection), hsa04217 (Necroptosis), and hsa05167 (Kaposi sarcoma-associated herpesvirus infection) (Fig. 8D, Table S5). The top five pathways were shown in Fig. 8E−I. These results suggested tight correlation of BLCA with virus infection.

## Discussion

BLCA is the second most common malignant urinary tumor with an increasing incidence all around the world [38]. The classic manifestation of BLCA patients is painless hematuria, which is usually evaluated with cystoscopy and upper tract imaging depending on the degree of hematuria and risk of malignancy [39]. With the rapid improvement of medical technology, the diagnosis and treatment of the disease have been obviously advanced but are still unsatisfied [40]. Therefore, it is of great clinical significance to clarify the molecular mechanism of BLCA for discovering new therapeutic strategies.

The highlight of the present study was the comprehensive analysis of OAS gene family potentially related to BLCA. The OAS gene family are associated with the occurrence and development of many diseases and pathologies, including chronic infections, autoimmune diseases [41], cancers [42], and COVID-19 [43]. Their functions include antiviral modulation [44], apoptosis [45], autophagy, receptor modulation, and signal transmission [46]. OAS gene family play an important role in COVID-19 due to their ability to activate the innate immune system against the coronavirus [43, 47]. Some studies suggest that OAS gene family may provide a potential therapeutic target for COVID-19 if the gene is mutated on chromosome 12q24.13 (a gene cluster that encodes OAS1, OAS2, and OAS3 antiviral restriction enzyme activators) [48]. Some other studies have suggested the relationship between OAS gene family and cancer, especially in breast cancer. For example, Zhang et al. [18] reported that high expression of OAS gene family is closely related to the occurrence of breast cancer, and OAS1 and OAS3 are correlated with poor prognosis of breast cancer. Lu et al. [49] explored the possible relations of lncRNA with the occurrence of breast cancer, and found that lncRNA TINCR can promote the proliferation and migration of breast cancer cells by regulating OAS1. Che et al. [50] also reported the association of OAS1 with breast cancer and found that OAS1 can regulate the resistance to tamoxifen. Gao et al. [51] reported the therapeutic value of OAS family in pancreatic cancer by analyzing the expression, prognostic value and biological function of OAS gene family in human pancreatic cancer. They demonstrated that OAS family members are all highly expressed in pancreatic cancer and may serve as biomarkers and therapeutic targets in pancreatic cancer. These studies suggest a tight association of OAS genes with cancer.

In human genome, the four OAS genes contain different numbers of OAS domain, which are coded by five exons. The OAS1 protein contains a single OAS domain, while the OAS2 and OAS3 proteins have two and three OAS domains, respectively. Human OASL protein contains an inactive OAS domain and two domains of ubiquitin-like sequences [52]. Among the four OAS genes, OAS1 has been particularly noticed. For example, Na et al. [53] observed that OAS1 is one of core genes related to the prognosis of bladder urothelial carcinoma via integrated bioinformatics analysis. Luo et al. [54] identified that OAS1 is one of the differentially expressed immune-related genes reflecting the microenvironment of BLCA based on TCGA and ImmPort databases. Here, we found that the expression of OAS1 in BLCA tissues was significantly higher than that in normal bladder tissues (Fig. 2), and the expression level was significantly high in clinical stages 1 − 4 compared to normal group (Fig. 3A). Through multiple databases analyses, we found that OAS1 was a clinically independent prognostic factor for BLCA, because the overall survival analyses via Kaplan–Meier plotter, Oncolnc and GEPIA databases all showed that OAS1 had positive effect on BLCA prognosis (Fig. 3B−D). In addition, researchers have developed some drugs targeting OAS1 to treat certain diseases, such as 5-azacytidine (AZA). As a DNA methyltransferase inhibitor, AZA can lead to tumor cell death through the 2’- 5’ oligoadenylate synthetase (OAS)-RNase L pathway. AZA has already been used as an approved drug in the treatment of myelodysplastic syndromes and acute myeloid leukemia. OAS1 expression is related to AZA sensitivity in the NCI-60 set of tumor cell lines, suggesting that the level of OAS1 can be a biomarker for predicting AZA sensitivity of tumor cells [55]. According to related studies, the expression level of OAS1 in anti-viral infection is higher than that of OAS2 and OAS3 [56], and there is a high correlation between the expression of OAS1 and the other three OAS genes, which may indicate the important role of OAS1.

OAS2 is one of antiviral interferon-stimulated gene and plays an important role in resisting virus infection [57] and innate immune response in COVID-19 [58]. OAS2 is related to many pathologies and diseases, including inflammation [59], autoimmune, malignant diseases, breast cancer [60], and colorectal cancer [61]. However, the biological functions of OAS2, including that in BLCA, remain to be clarified [62]. We showed here that the expression level of OAS2 in BLCA was higher than that in the normal bladder tissues (Fig. 2). To our knowledge, this is the first study to demonstrate the relationship between OAS2 and BLCA. OAS2 has outstanding performance in tumor immune cell infiltration (Fig. 4), but the specific mechanism remains to be identified.

OAS3 may also play a potential role in BLCA. The expression of OAS3 and other three OAS genes are closely related to each other, and their functions are similar. The 2',5'-oligoadenylate synthetase (OAS)-RNase L system is an antiviral signaling pathway induced by IFN, and OAS3 displays a higher affinity for dsRNA in intact cells than either OAS1 or OAS2 in the antiviral process, which is consistent with its dominant role in RNase L activation [63–65]. At present, only a few studies showed that OAS3 affects the occurrence and development of chronic lymphocytic leukemia [66, 67]. Unfortunately, till now, no study has been performed to show the function of OAS3 in BLCA. We showed here that the expression of OAS3 in BLCA was significantly higher than in normal bladder tissues (Fig. 2), suggesting the important role of OAS3 in BLCA. Not like the other three OAS genes that express in all species, OAS3 is only expressed in mice and humans. In addition, OAS3 does not harbor the catalytic activity required for synthesizing 2-5As and differs from the other human OAS family members by having two C-terminal ubiquitin-like domains. The detailed functional mechanisms of OAS3 in BLCA need further investigation.

Despite its lack of enzymatic activity, human OASL plays an important role in the antiviral process [68]. The relationship between OASL and cancer has been reported [69], such as breast cancer, cervical cancer, kidney cancer, and lung cancer. However, there is no report at present to show the role of OASL in BLCA. Here, we demonstrated that the expression of OASL was significantly higher in BLCA (Fig. 2) and had a beneficial effect on the overall survival based on analyses of Kaplan–Meier plotter and Oncolnc databases. Other studies have shown that OASL is closely related to the drug sensitivity of cervical cancer. Different expressions of OASL represent different drug-sensitivity to cisplatin in HPV + and HPV − cervical cancers. Patients with higher OASL expression exhibit stronger resistance to cisplatin than those with lower OASL expression [70]. OASL may also serve as a prognostic biomarker predicting the overall survival of Kidney Renal Clear Cell Carcinoma [71, 72]. Besides, OASL is closely related to the occurrence of lung cancer. So far, there have been studies on the important role of OASL gene in the treatment of lung cancer. Lv et al. [73] reported that OASL can be one of the decisive regulators to maintain lung cancer cell susceptibility to actinidia chinensis planch root extract and may be associated with the development of drug resistance. The regulation of OASL may be an alternative strategy to improve drug efficacy during cancer therapies.

The present study identified high expression of OAS gene family in BLCA and its association with some important biological processes of BLCA, including genetic and epigenetic alterations and immune cell infiltration, and identified its impact on BLCA prognosis. These findings suggest that OAS gene family is important in the pathogenesis and development of BLCA and may serve as biomarkers of this tumor. High expression of OAS family favors the survival and prognosis of BLCA patients. Thus, the OAS family may have clinical perspectives in the treatment of BLCA. For example, upregulating the expression of OAS family might have a therapeutic effect on BLCA.

The study has some limitations. First, we did not further experimentally validate the immune cells infiltration in the BLCA tissue which were shown in the bioinformatic analyses. Second, because of the molecular complexity of BLCA, we could not provide sufficient information on the systematic functions of OAS genes in the pathogenesis and progression of BLCA. These issues warrant future studies.

## Conclusion

The present study investigated the role of OAS gene family in BLCA, and found that high expression of OAS family favors the survival and prognosis of BLCA. The role of OAS1 in BLCA is particularly prominent among the four OAS genes. Several analytical results support the significance of OAS1 in BLCA: 1) OAS1 is not only highly expressed in BLCA tissues, but is also positively correlated with the prognosis of BLCA; 2) OAS1 exhibits larger fold change (logFC = 2.755) in expression than the other three OAS genes (Fig. 2A), both the mRNA and protein levels of OAS1 were higher than the other three OAS genes (Fig. 2C, D); 3) OAS1 can better predict the overall survival of BLCA patients from different databases (Fig. 3B). The study may contribute to better understanding on the molecular mechanisms and the target values of OAS gene family in BLCA.

## Supplementary Information

**Additional file 1: Table S1.** Characteristics of the patients diagnosed with carcinoma of urinary bladder. **Table S2.** Biological processes (BP) of GO analysis of top 20 genes closely related to OAS family. **Table S3.** Cellular components (CC) of GO analysis of top 20 genes closely related to OAS family. **Table S4.** Molecular Function (MF) of GO analysis of top 20 genes closely related to OAS family. **Table S5.** KEGG pathway analysis of top 20 genes closely related to OAS family.

**Additional file 2.**

## Data Availability

Oncomine (https://www.oncomine.org), Gene Expression Profiling Interactive Analysis (GEPIA, http://gepia.cancer-pku.cn/), Tumor Immune Estimation Resource (TIMER, https://cistrome.shinyapps.io/timer/), The Kaplan–Meier plotter (KM plotter, https://kmplot.com/analysis/), OncoLnc (http://www.oncolnc.org/), The cBio Cancer Genomics Portal (cBioPortal, https://www.cbioportal.org/), GeneMANIA (http://www.genemania.org), DAVID 6.8 (https://david.ncifcrf.gov/home.jsp), and KOBAS 3.0 (http://kobas.cbi.pku.edu.cn/anno_iden.php).
