# Supplementary material for: Values of OAS gene family in the expression signature, immune cell infiltration and prognosis of human bladder cancer
Source: BMC Cancer. 2022 Sep 26;22:1016. doi: 10.1186/s12885-022-10102-8 (PMC9510761; doi:10.1186/s12885-022-10102-8)
Supplement: Supplementary file 1 — Additional file 1: Table S1. Characteristics of the patients diagnosed with carcinoma of urinary bladder. Table S2. Biological processes (BP) of GO analysis of top 20 genes closely related to OAS family. Table S3. Cellular components (CC) of GO analysis of top 20 genes closely related to OAS family. Table S4. Molecular Function (MF) of GO analysis of top 20 genes closely related to OAS family. Table S5. KEGG pathway analysis of top 20 genes closely related to OAS family. [file 12885_2022_10102_MOESM1_ESM.docx]

**Values of** **OAS Gene Family in the** **Expression** **Signature,** **Immune Cell Infiltration and Prognosis of Human Bladder Cancer**

Lijuan Gao^1,2,^**^†^**, Ruimin Ren^3,^**^†^**, Jing Shen^1,2^, Jiayi Hou^4^, Junya Ning^1,2^, Yanlin Feng^1,2^, Meiyue Wang^1, 2^, Lifei Wu^1^, Yaojun Sun^1,2^, Huang Wang^3^, Deping Wang^1,2,*^, Jimin Cao^1,2,*^

^1^ Key Laboratory of Cellular Physiology, Ministry of Education, Shanxi Medical University, Taiyuan 030001, China

^2^ Department of Physiology, Shanxi Medical University, Taiyuan 030001, Shanxi Province, China

^3^ Department of Urology, Shanxi Bethune Hospital (Third Hospital of Shanxi Medical University), Taiyuan 030032, China

^4^ Department of Clinical Laboratory, Shanxi Provincial Academy of Traditional Chinese Medicine, Taiyuan 030012, China

**^†^** These authors contributed equally to this work.

*Correspondence:

Ji-Min Cao, MD, Department of Physiology, Shanxi Medical University, Taiyuan 030001, China. Email: caojimin@sxmu.edu.cn. ORCID: 0000-0002-6546-555X

De-Ping Wang, PhD. Department of Physiology, Shanxi Medical University, Taiyuan 030001, China. Email: wangdeping@sxmu.edu.cn. ORCID: [0000-0002-8479-5415](https://orcid.org/0000-0003-1445-4831)

**Table S1: Characteristics of the patients diagnosed with carcinoma of urinary bladder**

**Value**

Age(years) 65.7+10.1(mean+SD，n=7)

Sex:n(%) Male 7(100%),female 0(0%)

Tumor location and subtype(n) Posterior wall cancer of the bladder（1）

Carcinoma of left lateral wall of bladder（2）

Carcinoma of the right lateral wall of the bladder（1）

Carcinoma of the left anterior wall of the bladder（1）

Carcinoma of the left posterior wall of the bladder（1）

Carcinoma of posterior and lateral wall of bladder（1）

Pathological grading(n) T2N0M0（1） TaN0M0（6）

| **Table S2.** Biological processes (BP) of GO analysis of top 20 genes closely related to OAS family | | | |
| --- | --- | --- | --- |
| **ID** | **Term** | **P Value** | **Input Genes** |
| GO:0019985 | translesion synthesis | 0.048194 | ISG15, UBE2L6 |
| GO:0032480 | negative regulation of type I interferon production | 0.040319 | ISG15, UBE2L6 |
| GO:0006164 | purine nucleotide biosynthetic process | 0.016318 | OAS1, OAS2 |
| GO:0035457 | cellular response to interferon-alpha | 0.012263 | OAS1, IFIT3 |
| GO:0034340 | response to type I interferon | 0.00955 | MX1, ISG15 |
| GO:0032020 | ISG15-protein conjugation | 0.008191 | ISG15, UBE2L6 |
| GO:0019941 | modification-dependent protein catabolic process | 0.006831 | ISG15, UBE2L6 |
| GO:0060700 | regulation of ribonuclease activity | 0.002738 | OAS1, OAS3 |
| GO:0048285 | organelle fission | 0.002738 | MX2, MX1 |
| GO:0045087 | innate immune response | 2.47E-04 | BST2, MX2, MX1, IRF7, IFIT5, EIF2AK2 |
| GO:0035455 | response to interferon-alpha | 8.02E-05 | BST2, MX2, EIF2AK2 |
| GO:0035456 | response to interferon-beta | 6.42E-05 | BST2, STAT1, XAF1 |
| GO:0060333 | interferon-gamma-mediated signaling pathway | 4.39E-10 | OAS1, STAT1, OAS2, OAS3, IRF7, IRF9, OASL |
| GO:0045071 | negative regulation of viral genome replication | 2.34E-16 | BST2, RSAD2, OAS1, OAS3, MX1, EIF2AK2, ISG15, IFIT1, OASL |
| GO:0009615 | response to virus | 4.27E-21 | RSAD2, MX2, MX1, IFI44, EIF2AK2, IFIT1, IFIT3, OASL, BST2, OAS1, OAS2, OAS3, IRF7 |
| GO:0051607 | defense response to virus | 1.83E-25 | RSAD2, STAT1, MX2, MX1, IFIT5, EIF2AK2, ISG15, IFIT1, IFIT3, IFI44L, OASL, BST2, OAS1, OAS2, OAS3, IRF9 |
| GO:0060337 | type I interferon signaling pathway | 6.19E-35 | RSAD2, STAT1, MX2, MX1, ISG15, IFI35, IFIT1, IFIT3, OASL, BST2, IFI27, OAS1, OAS2, OAS3, IRF7, XAF1, IRF9 |

| **Table S3.** Cellular components (CC) of GO analysis of top 20 genes closely related to OAS family | | | |
| --- | --- | --- | --- |
| **ID** | **Term** | **P value** | **Input genes** |
| GO:0005829 | cytosol | 8.55E-07 | STAT1, MX2, MX1, EIF2AK2, ISG15, UBE2L6, IFI35, IFIT1, IFIT3, OASL, OAS1, OAS2, OAS3, IRF7, XAF1, IRF9 |
| GO:0005737 | cytoplasm | 0.00127 | STAT1, MX2, MX1, IFI44, EIF2AK2, IFIT1, IFIT3, IFI44L, OASL, BST2, OAS1, OAS2, OAS3, IRF7, IRF9 |
| GO:0005739 | mitochondrion | 0.02295 | IFI27, RSAD2, OAS1, OAS2, XAF1, IFIT3 |
| GO:0048471 | perinuclear region of cytoplasm | 0.042025 | STAT1, OAS2, MX1, EIF2AK2 |

| **Table S4.** Molecular Function (MF) of GO analysis of top 20 genes closely related to OAS family | | | |
| --- | --- | --- | --- |
| **ID** | **Term** | **P value** | **Input genes** |
| GO:0001730 | 2'-5'-oligoadenylate synthetase activity | 6.63E-09 | OAS1, OAS2, OAS3, OASL |
| GO:0003725 | double-stranded RNA binding | 8.82E-07 | OAS1, OAS2, OAS3, EIF2AK2, OASL |
| GO:0016740 | transferase activity | 2.20E-04 | OAS1, OAS2, OAS3, OASL |
| GO:0016779 | nucleotidyltransferase activity | 5.08E-04 | OAS1, OAS2, OAS3 |
| GO:0000975 | regulatory region DNA binding | 0.014831 | IRF7, IRF9 |
| GO:0005515 | protein binding | 0.021017 | RSAD2, STAT1, MX2, MX1, EIF2AK2, ISG15, UBE2L6, IFI35, IFIT1, IFIT3, BST2, OAS1, OAS2, OAS3, CHMP1A, IRF7, IRF9 |
| GO:0005524 | ATP binding | 0.033186 | OAS1, OAS2, OAS3, EIF2AK2, UBE2L6, OASL |

| **Table S5.** KEGG pathway analysis of top 20 genes closely related to OAS family | | | |
| --- | --- | --- | --- |
| **ID** | **Term** | **P-Value** | **Input Genes** |
| hsa05160 | Hepatitis C | 2.43E-18 | IRF7, STAT1, IRF9, EIF2AK2, MX1, OAS1, OAS3, OAS2, RSAD2, IFIT1 |
| hsa05164 | Influenza A | 7.33E-16 | IRF7, STAT1, IRF9, EIF2AK2, MX1, OAS1, OAS3, OAS2, RSAD2 |
| hsa05162 | Measles | 2.10E-14 | IRF7, STAT1, IRF9, EIF2AK2, MX1, OAS1, OAS3, OAS2 |
| hsa05169 | Epstein-Barr virus infection | 3.85E-13 | IRF7, STAT1, IRF9, EIF2AK2, OAS1, OAS3, OAS2, ISG15 |
| hsa05168 | Herpes simplex virus 1 infection | 4.02E-10 | IRF7, STAT1, IRF9, EIF2AK2, OAS1, OAS3, OAS2, BST2 |
| hsa04621 | NOD-like receptor signaling pathway | 1.22E-09 | IRF7, STAT1, IRF9, OAS1, OAS3, OAS2 |
| hsa05165 | Human papillomavirus infection | 4.44E-08 | STAT1, IRF9, EIF2AK2, OASL, MX1, ISG15 |
| hsa04217 | Necroptosis | 3.06E-06 | CHMP1A, EIF2AK2, IRF9, STAT1 |
| hsa05167 | Kaposi sarcoma-associated herpesvirus infection | 5.23E-06 | IRF9, EIF2AK2, IRF7, STAT1 |
| hsa05203 | Viral carcinogenesis | 0.000258 | IRF9, EIF2AK2, IRF7 |
| hsa04622 | RIG-I-like receptor signaling pathway | 0.000891 | IRF7, ISG15 |
| hsa04625 | C-type lectin receptor signaling pathway | 0.001916 | IRF9, STAT1 |
| hsa04620 | Toll-like receptor signaling pathway | 0.001916 | IRF7, STAT1 |
| hsa04380 | Osteoclast differentiation | 0.002862 | IRF9, STAT1 |
| hsa04630 | Jak-STAT signaling pathway | 0.004505 | IRF9, STAT1 |
| hsa05161 | Hepatitis B | 0.004558 | IRF7, STAT1 |
| hsa04623 | Cytosolic DNA-sensing pathway | 0.038402 | IRF7 |
| hsa05321 | Inflammatory bowel disease (IBD) | 0.039579 | STAT1 |
