# Supplementary material for: Values of OAS gene family in the expression signature, immune cell infiltration and prognosis of human bladder cancer
Source: BMC Cancer. 2022 Sep 26;22:1016. doi: 10.1186/s12885-022-10102-8 (PMC9510761; doi:10.1186/s12885-022-10102-8)
Supplement: Supplementary file 2 — Additional file 2. [file 12885_2022_10102_MOESM2_ESM.docx]

**Supplementary original western blot images**

OAS1-(N8, N9, N11, N12, T8, T9, T11, T12) for Fig.2D


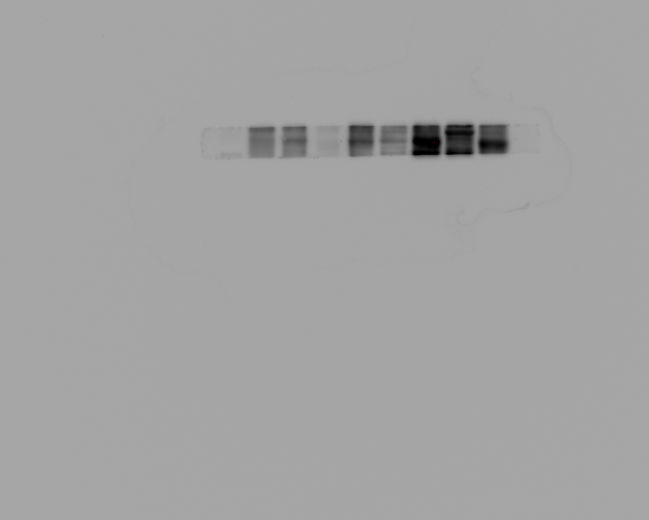


**N8 N9 N11 N12 T8 T9 T11 T12**

OAS1-(N2, N4, N6, T2, T4, T6) for Fig.2D


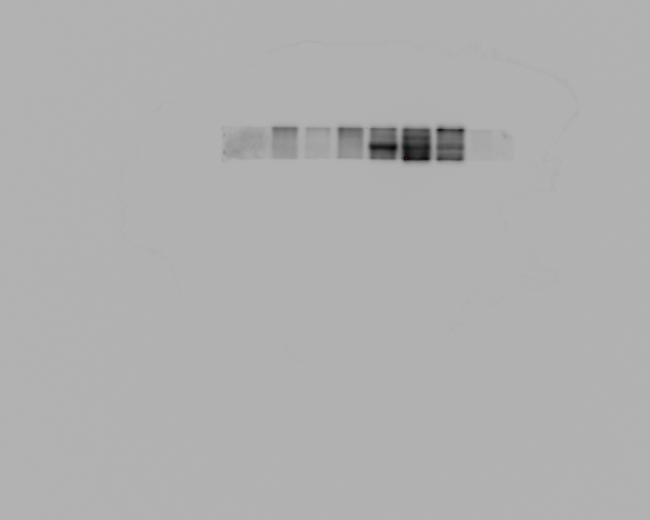


**N2 N4 N6 T2 T4 T6**

OAS1-β-actin-(N8, N9, N11, N12, T8, T9, T11, T12) for Fig.2D


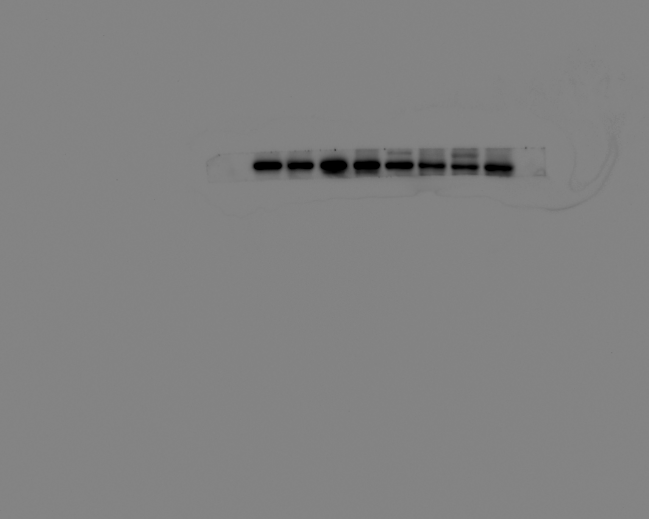


**N8 N9 N11 N12 T8 T9 T11 T12**

OAS1-β-actin-(N2, N4, N6, T2, T4, T6) for Fig.2D


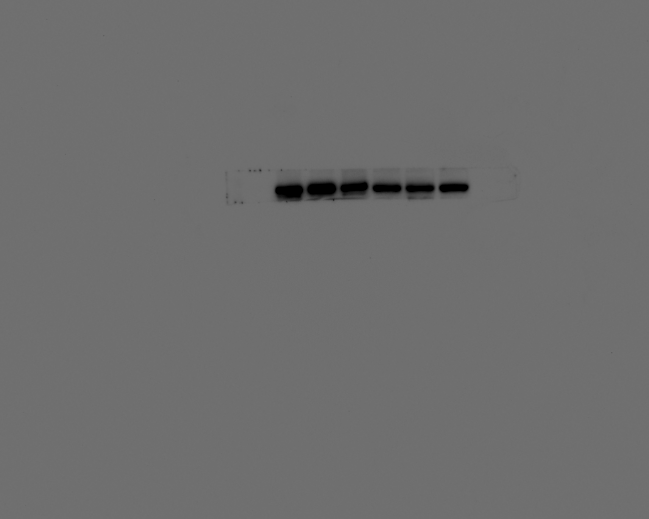


**N2 N4 N6 T2 T4 T6**

OAS2-(N8, N9, N11, N12, T8, T9, T11, T12) for Fig.2D

**
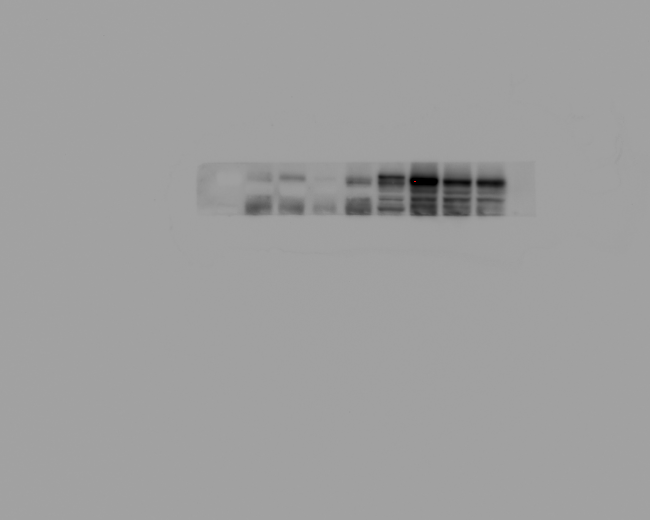
**

**N8 N9 N11 N12 T8 T9 T11 T12**

OAS2-(N2, N4, N6, T2, T4, T6) for Fig.2D

**
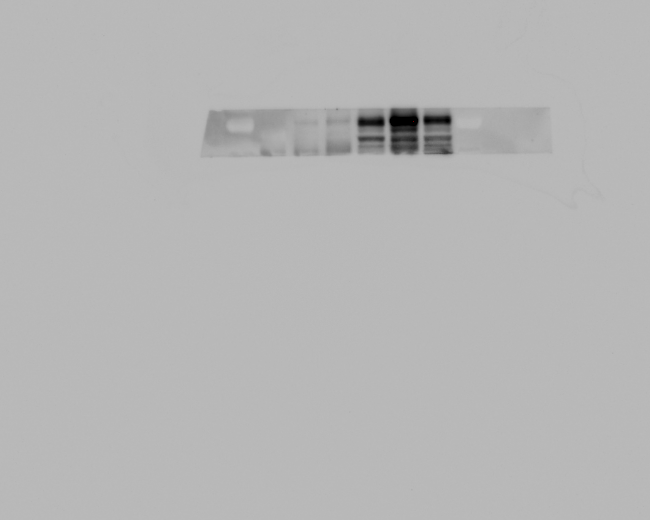
**

**N2 N4 N6 T2 T4 T6**

OAS2-β-actin-(N8, N9, N11, N12, T8, T9, T11, T12) for Fig.2D


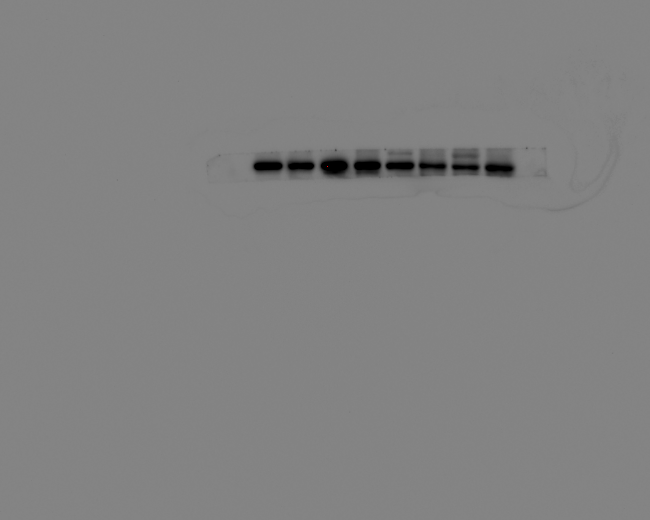


**N8 N9 N11 N12 T8 T9 T11 T12**


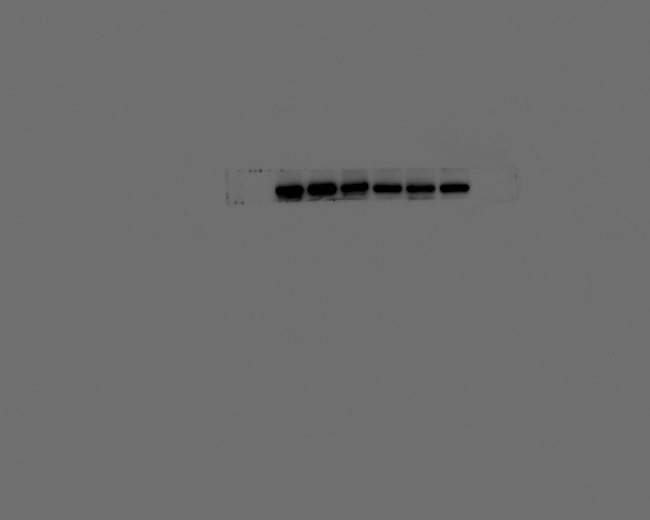
OAS2-β-actin -(N2, N4, N6, T2, T4, T6) for Fig.2D

**N2 N4 N6 T2 T4 T6**

OAS3-(N8, N9, N11, N12, T8, T9, T11, T12) for Fig.2D


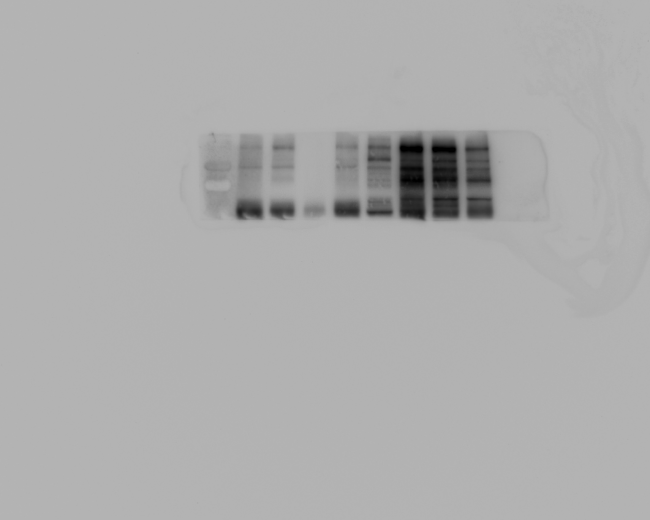


**N8 N9 N11 N12 T8 T9 T11 T12**

OAS3-(N2, N4, N6, T2, T4, T6) for Fig.2D
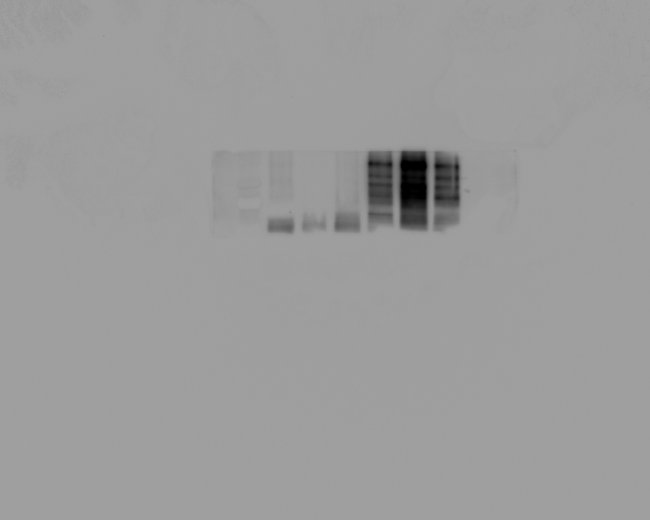


**N2 N4 N6 T2 T4 T6**

OAS3-β-actin -(N8, N9, N11, N12, T8, T9, T11, T12) for Fig.2D
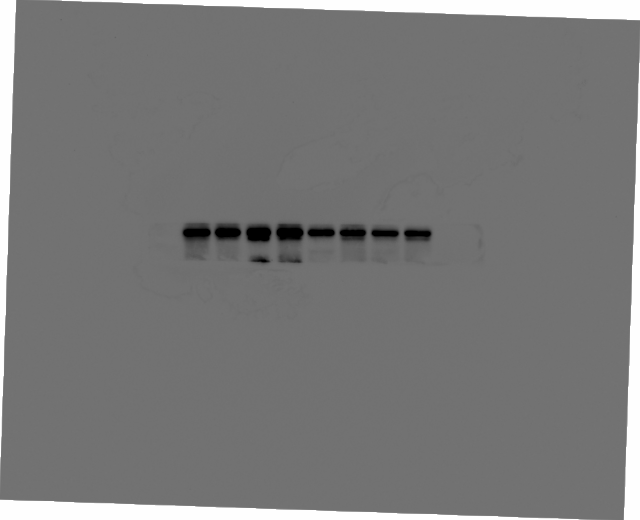


**N8 N9 N11 N12 T8 T9 T11 T12**

OAS3-β-actin -(N2, N4, N6, T2, T4, T6) for Fig.2D **
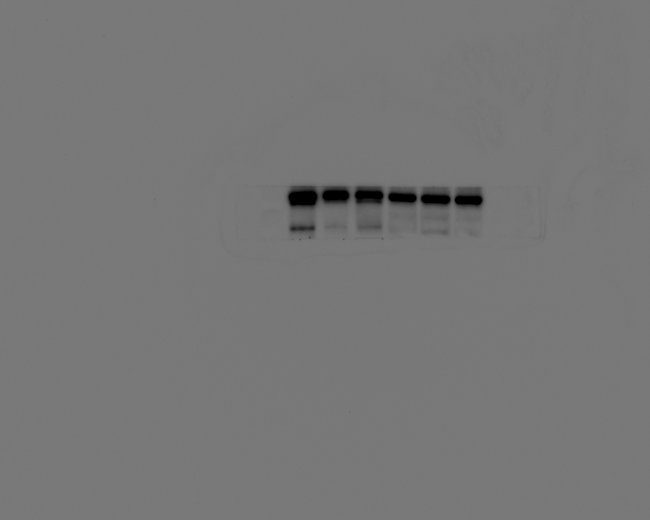
**

**N2 N4 N6 T2 T4 T6**

OASL-(N8, N9, N11, N12, T8, T9, T11, T12) for Fig.2D


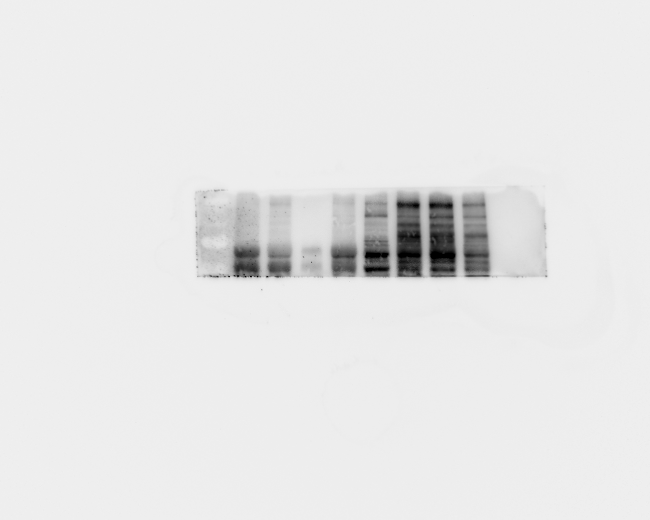


**N8 N9 N11 N12 T8 T9 T11 T12**

OASL-(N2, N4, N6, T2, T4, T6) for Fig.2D
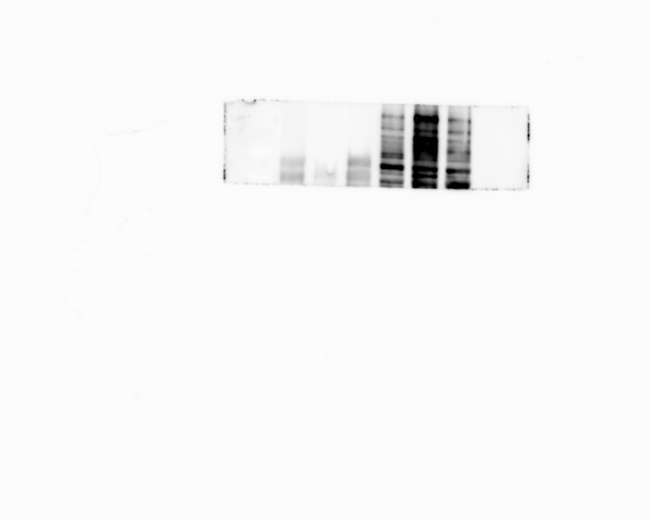


**N2 N4 N6 T2 T4 T6**

OASL-β-actin -(N8, N9, N11, N12, T8, T9, T11, T12) for Fig.2D
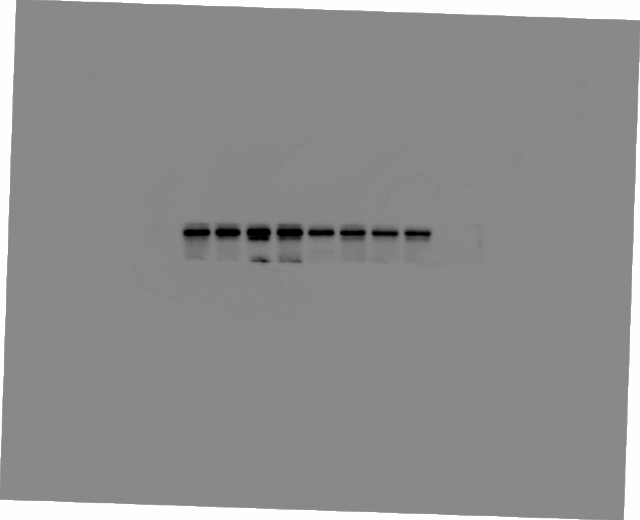


**N8 N9 N11 N12 T8 T9 T11 T12**

OASL-β-actin -(N2, N4, N6, T2, T4, T6) for Fig.2D
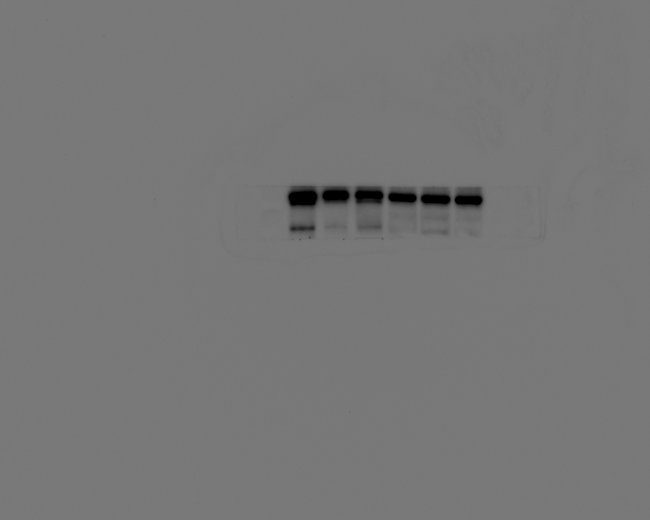


**N2 N4 N6 T2 T4 T6**
